# Commentary: Dog Stick Chewing: An Overlooked Instance of Tool Use?

**DOI:** 10.3389/fpsyg.2021.692495

**Published:** 2021-07-22

**Authors:** Ivaylo Borislavov Iotchev

**Affiliations:** Department of Ethology, ELTE Eötvös Loránd University, Budapest, Hungary

**Keywords:** tool use, dog cognition, cognitive evolution, comparative cognition, methods of behavioral research

Brooks and Yamamoto (2021) attempt to shed new light on old facts, when they propose that dogs' notorious chewing of sticks could be an overlooked instance of tool use. The arguments rely on an integrated analysis across many currently acknowledged definitions (Van Lawick-Goodall, 1971; Beck, 1980; Matsuzawa, 2008; Shumaker et al., 2011; Call, 2013) and propositions of how alternative accounts, such as the acts of playing or fidgeting relate to the proposed hypothesis. Brooks and Yamamoto synthetize and adopt a continuum-type hypothesis to support their final conclusion. This approach to theorizing acknowledges that higher cognitive functions might be absent in behaviors ancestral to tool use, but opens the door for other problems, to be elaborated below.

The authors' analysis avoids prioritizing among the existing definitions of tool use which leads to even deeper problems later on in the text. The middle section is a “dance” between proposing how alternate accounts could be controlled for, while also suggesting that playing, fidgeting, and tool use may not always be mutually exclusive. While this may do justice to the complexity of the topic it stands in the way of formulating a specific position relative to the problem. The final conclusion, to construct a continuum between the complete absence and presence of tool use and drop much of the cognitive expectations attached to the study of this behavior, may be justified by some of the existing definitions (Hall, 1963; Matsuzawa, 2008; Fragaszy and Mangalam, 2018), yet the approach runs into general problems to which I would like to dedicate the remainder of the commentary.

Compare the beginning of the piece “*Tool use is a central topic in research on cognitive evolution”* with its ending “*We instead emphasize […] that tool use as such can occur without positing complex reasoning abilities.”* Alas, a good theory is not complete by being provocative and testable. Answering it should teach us something new. That common, seemingly easy behaviors are actually very complex from, e.g., a neuro-computational perspective, is news only for the layman. The reason why tool use has accumulated the attention it deserves in comparative research, is because when sought for with stringent criteria it can allow us to explore the “upper limits” of the animal mind. A recent example is the use of tool manipulation to demonstrate long term planning in New Caledonian crows (Gruber et al., 2019).

Holding on to high standards when evaluating animal behaviors has been repeatedly advocated, both with regards to concluding tool use specifically (Sándor and Miklósi, 2020) and generally when studying non-human minds (Dawkins, 1993). Trying to avoid the efforts associated with these standards, by pruning the definition of tool use defeats the purpose of studying tool use. Therefore, to propose a widening of the definition in this manner, resembles a dog which tries to lift the extension of a mattress on which it is standing [but note, dogs do not do that (Lenkei et al., 2021)].

The continuum approach also promises something it cannot guarantee—that it can help us find a meaningful transition point between conscious tool use and similar (on the outside) motor behaviors. Within a continuum-framework, however, literally any act of movement could be reasoned an “ancestor” of tool use and this lack of selectivity and specificity does not advance the understanding of cognitive evolution or physical reasoning abilities. Put more simply: the idea of a continuum is not falsifiable. Furthermore, whether behavior A can evolve into behavior B could be constrained by the mechanisms underlying A, which is why a continuum starting on the level of appearances is likely misleading.

It is not the time to scrap self-awareness and intentionality from the definition of seemingly complex behaviors. Especially in dogs, a number of studies have demonstrated how far creative methodology can go in reaching for the high-hanging fruit. The do-as-I-do training method, originally introduced by Topál et al. (2006), utilizes the animals' imitation capacity and complex training regimes to tackle tricky questions. This method alone has come a long way. Fugazza et al. (2016, 2020) used it to argue episodic memories and awareness of own actions in the dog. More recently, and as humorously referenced above, dogs were tested for grasping that their own body can be an obstacle in their interaction with the world (Lenkei et al., 2021). This study, in which dogs were rewarded for lifting objects off the ground, showed that the animals readily stepped down from a mattress on which they were standing, if the object to be lifted was attached to it. Otherwise unmovable objects did not elicit the same response, strongly suggesting that the behavior was guided by a “representation” of how the dogs' body was related to its surrounding and goals. There are also known limitations to dogs' capacity for physical reasoning (Müller et al., 2011; Lampe et al., 2017), yet the increasing number of paradigms invented or adapted for dogs, invite bolder questions and not simpler definitions.

Ultimately, our attempts to come up with new research efforts might indeed benefit from shaking up rigid definitions, but we need to keep an eye on the bigger picture when doing so. Being flexible about the definition of what counts as a tool is actually a good implication to extract from Brooks and Yamamoto (2021). Some forms of communication may count, if we allow the tool to be, itself, an agent. In as far as dogs' “showing behavior” (Miklósi et al., 2000) is intending to overcome obstacles by “asking help” from another agent (the behavior is exhibited toward humans, when preferred objects are out of reach) this could be an overlooked instance of tool use. Note, however, that we should and cannot be satisfied with “black boxes.” Comparing only the overt expression of human and animal behavior is the “easy way out” and in the worst case leads to premature and likely false equivalences [e.g. seen in Chapman and Huffman (2018)].

One of the great empirical challenges we face now, when it comes to tool use, is to have combined experiments that simultaneously probe aspects of self-awareness and intention on one hand, and complicated manipulations of objects, other agents or the environment on the other. As seen by the example literature provided above, each by itself can be approximated to a reasonable degree in dogs, thus the right step forward is to design paradigms that combine these approaches toward a holistic understanding of behavior encompassing both the function, but also the underlying intentions and representations. Concerning the latter, the methods of neuroscience, their limitation noted, can provide useful auxiliary arguments when the implications of measurable behavior remain vague. Note for example how the study of place-cell activation in the rat hippocampus, has produced arguments for the animals' capacity to represent their navigation experiences as re-playable sequences (Karlsson and Frank, 2009). The latter is at least a functional analog of complex mental representations, be it that whether these processes are sentient or even conscious will be hard to judge given the unresolved issues in the “Philosophy of Mind” field (reviewed in Blackmore, 2005; Ravenscroft, 2005).

In conclusion, being flexible about the definition of a tool is a welcome suggestion, but being flexible about the mental processes and representations inherent to tool use is not a good idea. Mapping the difference and overlap between different minds is an exciting endeavor within comparative cognition, but it depends upon solid and non-negotiable definitions of what constitutes a given mental process. And that's the crucial point. Tool use is not just a behavior. Our interest in tool use is due to the implied underlying mentalization and therefore dropping this from its definition is not open to negotiation.

## Author Contributions

The author confirms being the sole contributor of this work and has approved it for publication.

## Conflict of Interest

The author declares that the research was conducted in the absence of any commercial or financial relationships that could be construed as a potential conflict of interest.
